# Methods for Quantitative Determination of Iron Sulfides in Rocks

**DOI:** 10.3390/ma18112647

**Published:** 2025-06-05

**Authors:** Zhixin Wang, Shaoping Wang, Wei Li, Bing Cao, Xiaojun Huang, Xin Chuai, Xinyu Zhang, Min Deng

**Affiliations:** 1 College of Materials Science and Engineering, Nanjing Tech University, Nanjing 211816, China; 202261203229@njtech.edu.cn (Z.W.);; 2 Nanshan Mining Company Ltd., Anhui Maanshan Iron and Steel Mining Resources Group, Maanshan 243000, China

**Keywords:** pyrite, selective dissolution, quantitative analysis, aggregate

## Abstract

When iron sulfides are used as aggregate in concrete production, it easily oxidizes to form harmful substances such as sulfates. This results in acid corrosion and internal sulfate attack (ISA), significantly reducing concrete durability. To date, the quantification methods for iron sulfides in aggregates remain inaccurate, often neglecting pyrrhotite (a type of iron sulfide). No standardized methods or threshold values for the sulfide content in aggregates have been established, nor have technical guidelines for the application of sulfide-containing aggregates, limiting their use. This study proposes an on-site quantification procedure for determining the pyrite and pyrrhotite content in tailings using a selective chemical dissolution process. An orthogonal experiment was designed to determine the optimal dissolution conditions by considering four factors: particle size, reaction temperature, acid concentration, and reaction time. The pyrrhotite quantification method showed a relative standard deviation (RSD) of 3.60% (<5%) and a mean relative error of 3.19% (<5%), while the pyrite quantification method showed an RSD of 3.11% (<5%) with a mean relative error of 4.70% (<5%). The results were further optimized under engineering conditions to reduce costs and enable on-site quantification without relying on complex precision instruments. The quantitative results of pyrite in mineral samples were verified by the XRD internal standard method, and the error was less than 0.6%. This approach ensures the effective monitoring and management of sulfide content in concrete aggregates, promoting the practical application of sulfur-bearing aggregates.

## 1. Introduction

In the 20th century, sulfide-containing aggregates (mostly from tailings) were widely used in concrete in Europe and America to reduce the construction costs [1,2,3,4]. These rocks contained sulfides like pyrite, which later oxidized, causing expansion and cracking, and progressively deteriorated the concrete [5,6,7,8]. In Massachusetts, eastern U.S., extensive cracking damage has occurred in numerous residential concrete foundations due to the oxidation of iron sulfide minerals in coarse aggregate stone, affecting over 45,000 households [9]. In Spain, the use of pyrite-containing aggregates in construction has led to numerous cases of oxidation-induced structural damage [10]. In 1992, iron-sulfide-bearing aggregates are used in the Grosse and Taiscon dams, which subsequently led to severe concrete cracking problems. Since 2004, concrete expansion and rapid deterioration have been observed within 2 to 5 years after construction in Trois-Rivières, Quebec, Canada [11,12]. In eastern Connecticut, USA, numerous homes incorporating pyrrhotite-bearing aggregates have exhibited cracking and structural damage after 25 years of service [13].

Metal sulfide tailings exposed to air undergo oxidation over time, generating multiple hazardous substances that cause severe environmental damage [6,14,15,16,17]. Metal sulfides in mine tailings are highly reactive. Pyrite (FeS_2_), in particular, easily reacts with oxygen when exposed to air or oxygen-containing water [18,19]. In wet conditions, pyrite slowly dissolves, releasing large amounts of H^+^. At the same time, heavy metals like Zn^2+^, Cu^2+^, Cd^2+^, and Pb^2+^ inside the mineral also dissolve out [20]. It is most accepted that the process mainly includes the following steps [21,22,23]:

When exposed to the environment, pyrite reacts with water and oxygen, producing Fe^2+^ and SO_4_^2−^, and releasing large amounts of H^+^, as shown in Equation (1).

2FeS_2_ + 7O_2_ + 2H_2_O → 2Fe^2+^ + 4H^+^ + 4SO_4_^2−^ (1)

The generated Fe^2+^ is unstable in the environment and is rapidly oxidized by oxygen to Fe^3+^, as shown in Equation (2).

4Fe^2+^ + O_2_ + 4H^+^ → 4Fe^3+^ + 2H_2_O (2)

Due to the substantial H^+^ production from Equation (1), the environmental acidity progressively increases. When the pH falls below 3.5, Fe^3+^ acts as a potent oxidant to further oxidize pyrite, as shown in Equation (3).

FeS_2_ + 14Fe^3+^ + 8H_2_O → 15Fe^2+^ + 16H^+^ + 2SO_4_^2−^ (3)

The Fe^2+^ produced in Equation (3) reacts with environmental oxygen via Equation (2), forming the highly oxidative Fe^3+^. This cyclic process leads to the continuous accumulation of Fe^3+^ and SO_4_^2−^, accompanied by the progressive acidification (pH decrease) of the environment [24].

The use of iron sulfides as an aggregate in concrete poses risks due to oxidation and sulfate formation. The accurate quantification of iron sulfides content is essential in order to ensure concrete durability.

Luo conducted a qualitative and quantitative analysis of pyrite materials using analytical transmission electron microscopy (AEM/EDS), which enables the precise quantitative analysis of micron- and nano-scale regions in pyrite materials [25]. McDougall, H. conducted a quantitative phase analysis of pyrite concentrate powder using X-ray diffraction [26]. Del Real I proposed a method for the accurate quantification of pyrite using synchrotron-based X-ray fluorescence [27]. The study by Bolin, TB quantified the pyrite content by combining the sulfur K-edge X-ray absorption near-edge structure (S-XANES) third-derivative analysis with wet chemical methods [28].

These methods all require expensive and sophisticated instrumentation, making them unsuitable for a reproducible on-site quantitative analysis. Alternatively, some standard methods employ acid dissolution techniques, where the soluble sulfur content is assumed to represent the pyrite content. The national standard GB/T 31288 [29] establishes the technical requirements for iron tailings sand used in concrete, concrete products and mortar. It specifies that the sulfide and sulfate content (calculated as SO_3_) in iron tailings sand should not exceed 0.50%. This method specifies dissolving the sample in HCl, followed by the determination of the dissolved SO_4_^2−^ content using the barium chloride titration method. The obtained SO_4_^2−^ concentration is then converted to SO_3_ content, representing the total sulfides and sulfates in the sand. However, since pyrite is insoluble in HCl, this method cannot evaluate the potential hazards from sulfur present in pyrite form, representing a significant limitation in assessing the complete sulfur-related risks of iron tailings sand.

The European standard EN 12620:2008 [30] specifies that the pyrrhotite content in aggregates, calculated as sulfur, must not exceed 0.1%. Currently, no other countries or regions have established limit standards for the iron sulfide mineral content in aggregates. Similarly, ACI 201.2R [31] does not provide specific regulations on this matter.

Jana [13] found that Canadian aggregates containing 0.3% pyrrhotite caused severe concrete cracking. To mitigate this, they recommend either avoiding pyrrhotite-containing aggregates, using aggregates with a sulfur content below 0.1%, or establishing sulfur threshold limits specific to different iron sulfide mineral types.

The oxidation of pyrrhotite is more significant than that of pyrite due to its higher reactivity. Existing methods primarily focus on the quantification of pyrite, lacking the ability to distinguish and identify pyrite and pyrrhotite. Furthermore, some standards fail to effectively quantify pyrite, highlighting the need for improved analytical approaches.

This study aims to determine the content of sulfide minerals (including pyrite and pyrrhotite) in tailings using a stepwise chemical dissolution method. An HCl solution was used to selectively dissolve pyrrhotite first, followed by an HNO_3_ solution to dissolve pyrite due to its higher oxidizing power. The results were validated by a quantitative analysis using XRD. The effects of the sample particle size, solution concentration, reaction temperature, and reaction time on the analysis of the sulfide mineral content will be examined to establish suitable sample sizes and experimental conditions.

## 2. Materials and Methods

### 2.1. Materials

#### 2.1.1. Pyrrhotite

**Figure 1:**
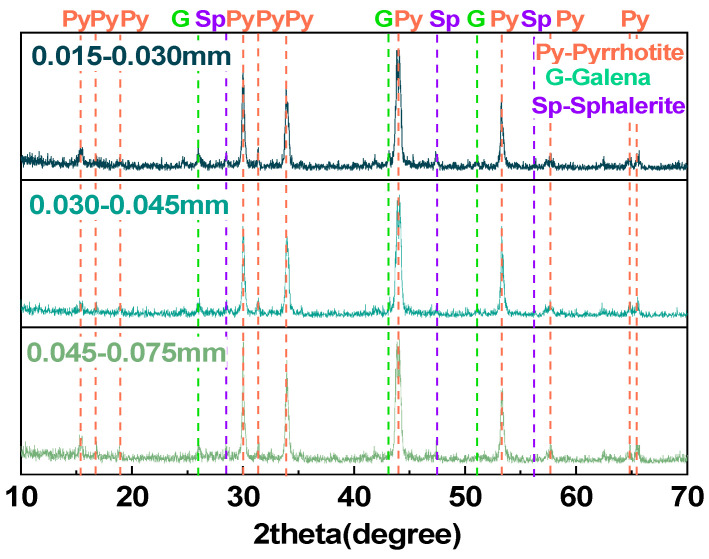
XRD patterns of pyrrhotite in 0.015–0.030 mm, 0.030–0.045 mm, and 0.045–0.075 mm.

The pyrrhotite samples used in this experiment are collected from the Huanggangliang iron mine in Chifeng, Inner Mongolia, China. After crushing and sieving, the samples were classified into three particle size fractions: 0.015–0.030 mm, 0.030–0.045 mm, and 0.045–0.075 mm. Table 1 shows the elemental compositions of different size of pyrrhotite with Thermo ARL 9900 X-ray fluorescence (XRF), from Thermo, Waltham, MA, USA. The pyrrhotite with three different particle sizes are mainly composed of Fe and S, with trace amounts of impurity elements like Pb, Zn, and Si. Figure 1 shows distinct characteristic diffraction peaks of pyrrhotite (Fe_7_S_8_), galena (PbS), and sphalerite (ZnS). The X-ray powder diffraction data were collected at room temperature using a Rigaku SmartLab 3 kW diffractometer, from Rigaku, Tokyo, Japan.

#### 2.1.2. Pyrite

**Figure 2:**
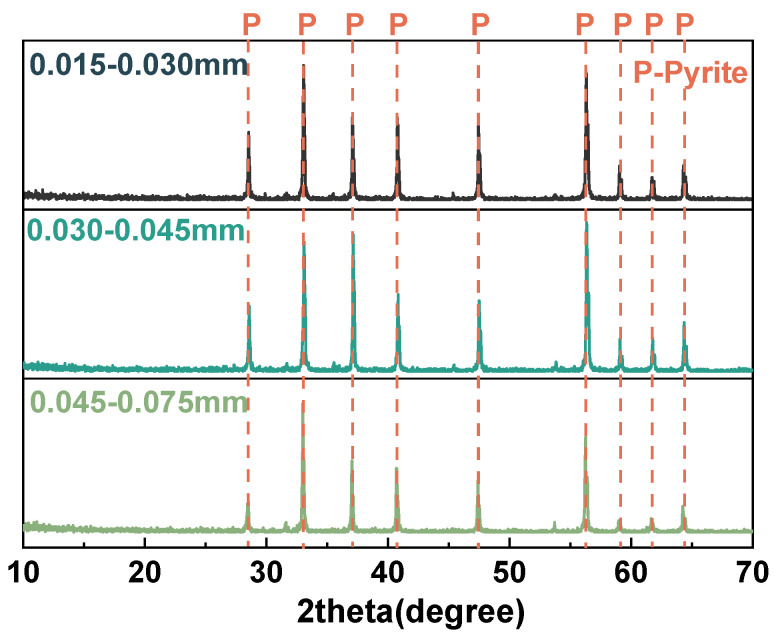
XRD patterns of pyrite in 0.015–0.030 mm, 0.030–0.045 mm, and 0.045–0.075 mm.

The pyrite samples used in this experiment are collected from the Shangbao mining area in Hunan, China. After crushing and sieving, the samples were classified into three particle size fractions: 0.015–0.030 mm, 0.030–0.045 mm, and 0.045–0.075 mm. Table 2 shows the elemental compositions of different sizes of pyrite. The pyrite with three different particle sizes is mainly composed of Fe and S. Figure 2 shows distinct characteristic diffraction peaks of pyrite.

#### 2.1.3. Iron Ore Tailings

**Figure 3:**
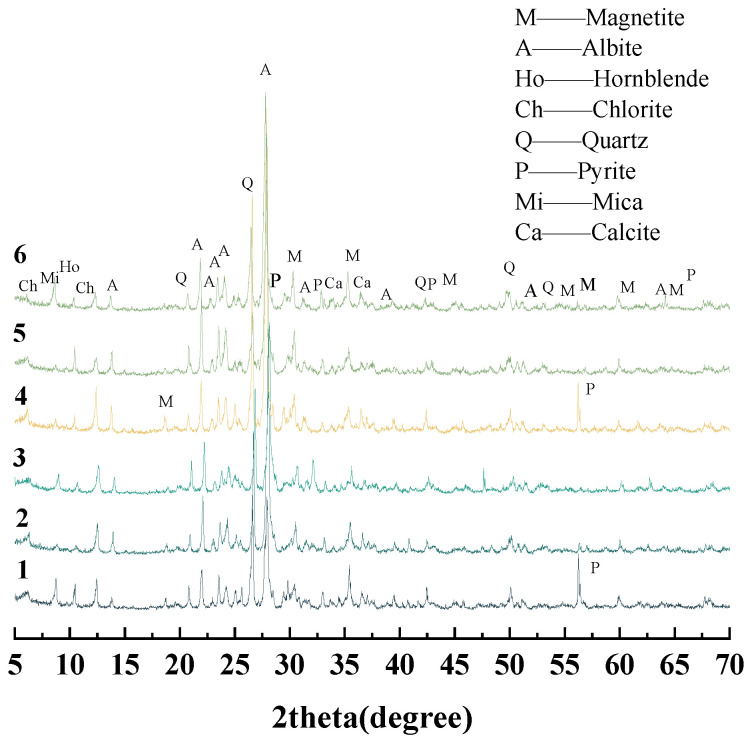
XRD patterns of sample 1–6.

**Figure 4:**
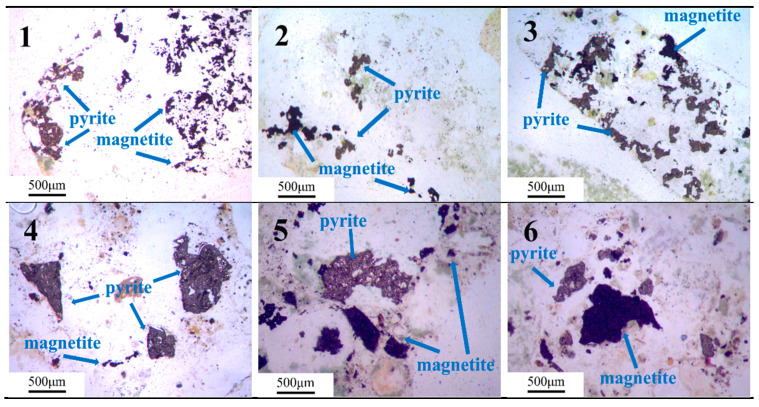
Micrographs of samples 1–6.

Samples numbered 1–3 are the crushed stone, while samples numbered 4–6 are the manufactured sand. There samples are collected from the maanshan iron and steel mining resources group in Anhui, China. The blended samples are ground using a vibratory mill to ensure they passed through a 0.075 mm standard sieve. Figure 3 shows that Samples 1–6 are primarily composed of feldspar, with minor amounts of amphibole, chlorite, mica, calcite, quartz, magnetite, and pyrite. Figure 4 shows the micrograph obtained by optical microscope. Pyrite is dispersed throughout the sample, locally exhibiting a mosaic texture, with minor amounts of pyrite coexisting with magnetite.

### 2.2. Methods and Device

#### 2.2.1. Chemical Dissolution Method

**Figure 5:**
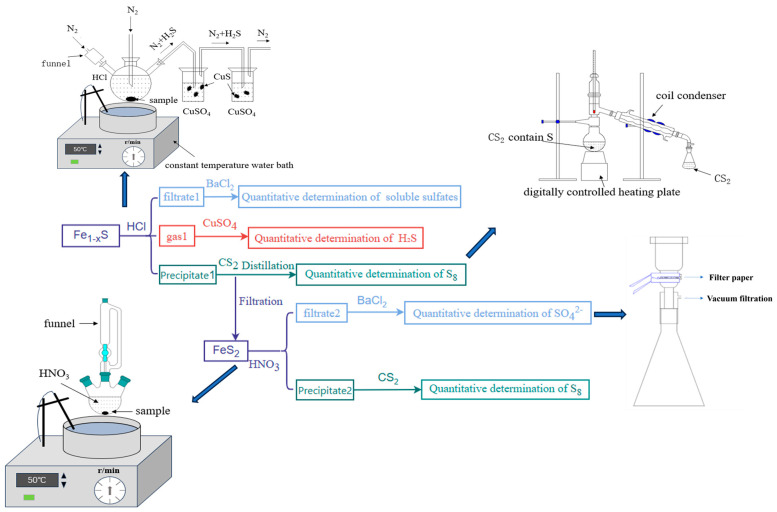
Experimental procedure and equipment diagram.

In the presence of HCl solutions, pyrrhotite can undergo complete dissolution, resulting in the production of H_2_S and elemental sulfur. H_2_S-containing gas is absorbed by CuSO_4_ solution, forming CuS precipitate. The amount of H_2_S is quantified by measuring the precipitate. Elemental sulfur, which is highly soluble in carbon disulfide, can be quantified by evaporating carbon disulfide. By measuring these two sulfur-containing products, the sulfur content in the pyrrhotite sample can be accurately quantified. The value of x in pyrrhotite was determined by X-ray diffraction analysis to calculate the pyrrhotite content in the sample. At the same time, the filtrate after the reaction can be analyzed for soluble sulfate content (as SO_3_ mass fraction). (As shown in Figure 5).

The experimental procedures are as follows:

1. The pyrrhotite was added to HCl, and the evolved gas was purified using two gas-washing bottles containing CuSO_4_ solution.
2. After the reaction is complete, the reaction mixture is filtered. The filtrate (Filtrate 1) is added with BaCl_2_ to form precipitate BaSO_4_, which allows for the quantitative determination of soluble sulfate content.
3. While the filter precipitate (precipitate 1) was dried, the dried precipitate 1 was then washed with CS_2_, followed by filtration. The solution was evaporated to recover elemental sulfur.
4. The precipitate in the two gas-washing bottles is filtered, dried, and weighed to obtain the mass of CuS.
5. The presence of sphalerite and galena as impurities in the pyrrhotite ore introduces analytical interference, as these minerals are also soluble in HCl. This may affect the accuracy of sulfur quantification. To quantitatively analyze the Pb^2+^ and Zn^2+^ ion concentrations in the sample, H_2_SO_4_ is added to the filtrate to precipitate Pb^2+^ ions as PbSO_4_. The resulting PbSO_4_ precipitate is dissolved in a pH 5.7 buffer solution (acetic acid/sodium acetate), and the lead content is determined via EDTA complexometric titration. In an ammoniacal medium with the presence of an oxidizing agent, Fe^2+^ interference is precipitated and separated. Na_2_S_2_O_3_ and NH_4_F are used as masking agents to eliminate the interference from Cu^2+^ and Al^3+^. Zn^2+^ content is determined by EDTA standard solution titration at pH 5.5–6.0, using xylenol orange as the indicator.

**Figure 6:**
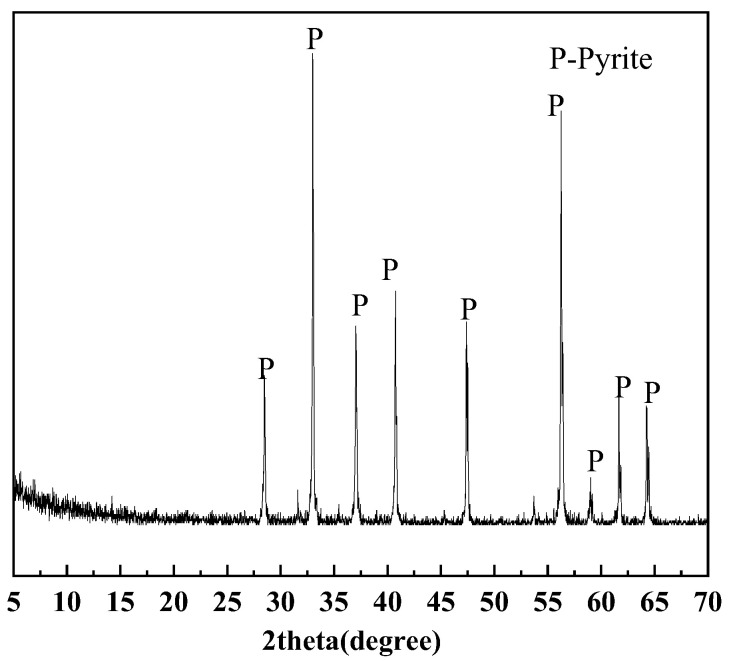
XRD pattern of the reaction product from pyrite treated with 1:1 HCl solution at 80 °C for 1 h.

Unlike pyrrhotite, pyrite has a more stable crystal structure and does not dissolve in HCl solution. Figure 6 shows the X-ray diffraction pattern of the reaction product from pyrite and HCl. However, it can dissolve completely in strongly oxidizing HNO_3_ solution. During the dissolution of pyrite, sulfur in pyrite is oxidized to SO_4_^2−^ ions and partially to elemental sulfur. The SO_4_^2−^ ions in the solution can be quantified by adding saturated BaCl_2_ solution to form BaSO_4_ precipitate. Elemental sulfur is dissolved in CS_2_ and recovered through distillation for quantitative analysis. By combining the measured masses of SO_4_^2−^ ions and elemental sulfur, the sulfur content in the pyrite can be quantified. The pyrite content in the sample was quantified based on its stoichiometric chemical composition. (As shown in Figure 5).

The experimental procedures are as follows:

1. The pyrite was added to HNO_3_ solution.
2. After the reaction was completed, the mixture was filtered through filter paper to obtain precipitate 2, while filtrate 2 was diluted to 100 mL. BaCl_2_ solution was added to filtrate 2 to quantitatively precipitate SO_4_^2−^ ions.
3. The remaining precipitate was dried, washed with carbon disulfide, and filtered. The resulting solution was then distilled to recover elemental sulfur.

#### 2.2.2. The Internal Standard Method of XRD

**Figure 7:**
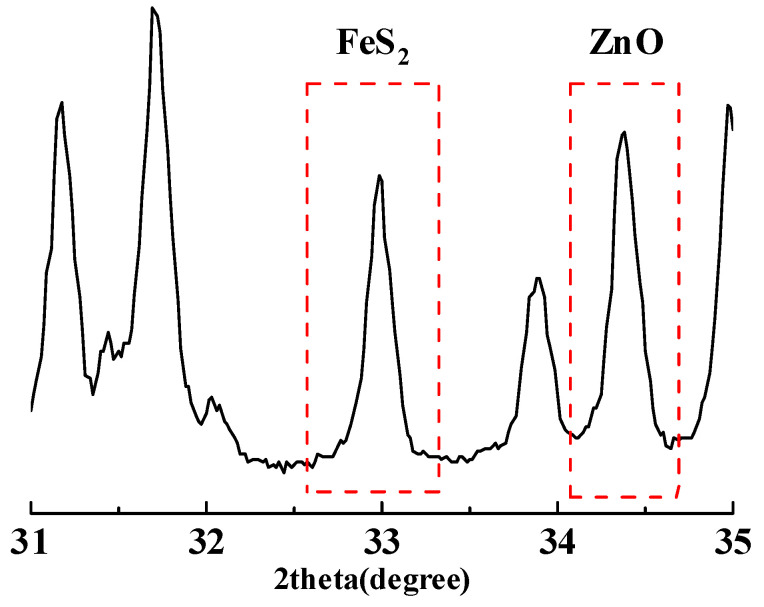
Diffraction peaks of pyrite and ZnO.

**Figure 8:**
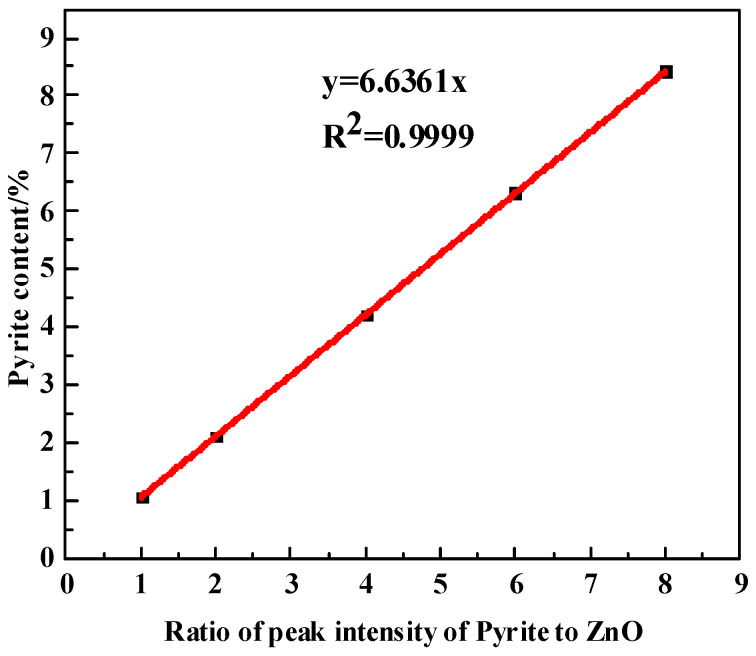
The standard working curve of the internal standard method.

The samples were completely crushed and processed into crushed stone with particle sizes of 5–25 mm and sand with a fineness modulus of approximately 2.80. All samples were thoroughly mixed and approximately 30–50 g of the samples taken and pulverized using a vibrating mill for later use. Quantitative analysis of pyrite in crushed stone and sand samples was conducted using the XRD internal standard method, with analytical-grade ZnO as the internal standard. A 2.0 g mixed sample was prepared by mixing according to the proportions presented in Table 3 and ground in an agate mortar for 20 min. The samples were tested in the 31~35° range with a scanning speed of 0.02°/s. Figure 7 shows the X-ray diffraction pattern of pyrite and ZnO. Figure 8 shows the areas of pyrite and ZnO were calculated using the MDI Jade 6 software for fitting, and the resulting calibration curve.

### 2.3. Orthogonal Design of Experiments

Four main factors (particle size, reaction temperature, reaction concentration, and reaction time) are each set at three levels. Each combination was tested in triplicate. Two L9(3^4^) orthogonal tables which are presented in Table 4 and Table 5 were employed to conduct experiments for optimizing quantitative conditions, ultimately determining the best process conditions for quantifying iron sulfide in the samples.

## 3. Results and Discussion

### 3.1. Quantification of Pyrrhotite

Under the nine experimental conditions of the orthogonal design table, approximately 0.5 g of the sample was weighed (accuracy: ±0.0001 g) and reacted with 50 mL of acids (HCl) at varying concentrations. The evolved gases were purified by passing through two gas-washing bottles, each containing 500 mL of 1 mol/L CuSO_4_ solution.

The Pb^2+^ and Zn^2+^ in the filtrate are quantitatively analyzed. Subsequently, corrections are made for their contributions based on the chemical formulae of PbS and ZnS.

The pyrrhotite content was quantified based on the calculated mass of sulfur in individual components, as shown in Equations (4) and (5).

(4) $$m_{total\;S}=m_{elemental\;S}+m_{Sin\;H_{2}S}-m_{Sin\;PbS}-m_{Sin\;ZnS}$$

(5) $$w_{{Fe}_{7}S_{8}}=\frac{m_{total\;S}\times 2.61}{m_{sample}}$$

where $M_{{Fe}_{7}S_{8}}=669.23\;g/mol$ and $M_{S}=32.06\;g/mol$. This yields a conversion factor of 2.61 for transforming the sulfur mass to a pyrrhotite mass.

Table 6 shows the quantitative analysis results of sulfur-containing products. The analysis of pyrrhotite in the sample was performed based on its chemical formula Fe_7_S_8_, with the sulfur content calculated accordingly.

**Figure 9:**
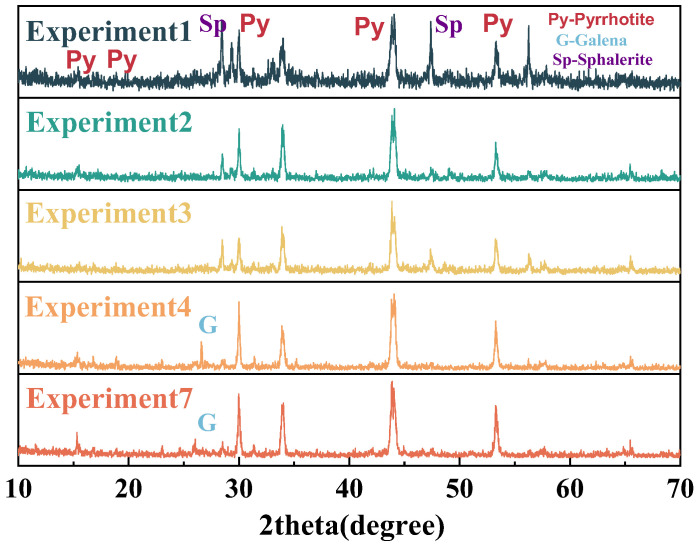
Mineral composition of HCl acid insoluble residues of pyrrhotite.

Figure 9 shows the presence of unreacted pyrrhotite in the X-ray diffraction pattern. In experiments 4 and 7, no precipitate was observed in the gas-washing bottle, showing no H_2_S gas was generated. Adding a BaCl_2_ solution to the filtrate produced no white precipitate, indicating no SO_4_^2−^ ions were formed. The CS_2_ dissolved and no elemental sulfur was detected after distillation. Therefore, pyrrhotite in experiments 4 and 7 did not react with HCl. The reaction rates of pyrrhotite in experiments 1, 2, and 3 were 33.15%, 26.41%, and 72.91%, respectively. The residue contained unreacted pyrrhotite and sphalerite, indicating that the reaction between pyrrhotite and hydrochloric acid was incomplete under the particle size range of 0.015–0.030 mm.

The concentration of the reference value in Table 7 was determined by an XRF analysis and calculated after excluding the interference of sulfur from the associated galena and sphalerite phases.

Table 7 shows the following significance ranking of four influencing factors: R2 > R3 > R4 > R1. This indicates that the reaction temperature exerts the most substantial effect on the pyrrhotite quantification, followed by the solution concentration, reaction time, and particle size. The optimal conditions are determined to be A3B3C1D1, corresponding to a particle size of 0.045–0.075 mm, reaction temperature of 80 °C, HCl concentration of 1:1, and reaction time of 1.5h.

Besides the optimization plan, the results also indicate that, at a temperature range of 50–80 °C, particle sizes of 0.030–0.075 mm, a reaction time of over 1.5 h, and an HCl concentration exceeding 25%, the quantitative analysis of pyrrhotite becomes more accurate. However, when measuring tailings samples, the particle size distribution may not be as distinct. Therefore, it is necessary to test whether the optimal experimental conditions remain reasonable under a particle size range of below 0.075 mm and make further optimizations accordingly. The ratio in the quantitative results under the 1:1 HCl solution condition in Table 7 is significantly smaller, so the adjustment of the reaction temperature is preferred when optimizing the conditions.

**Figure 10:**
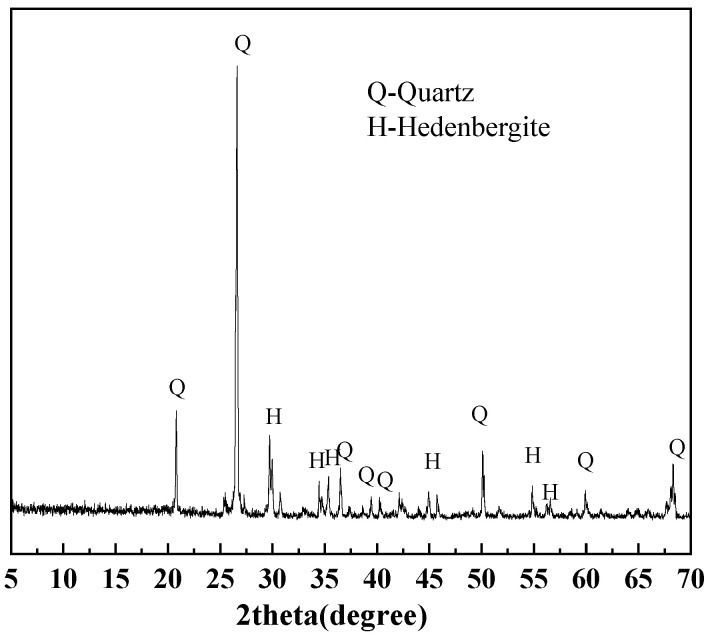
XRD pattern of pyrrhotite reacted with 1:1 HCl solution at 80 °C for 1.5 h.

The pyrrhotite sample is dissolved in the 1:1 HCl solution at 80 °C for 1.5 h. Figure 10 shows no detectable pyrrhotite diffraction peaks, confirming complete dissolution under these conditions.

**Figure 11:**
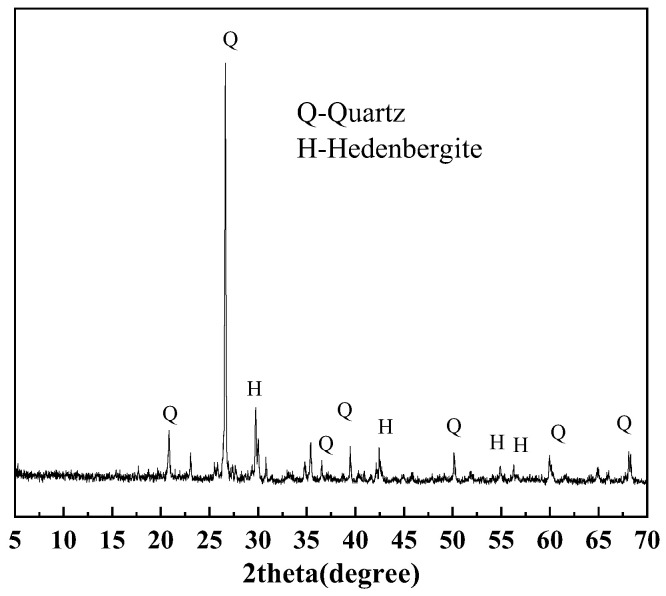
XRD pattern of pyrrhotite reacting with 1:1 HCl solution at 50 °C for 1.5 h.

Pyrrhotite is dissolved in a 1:1 HCl solution at 50 °C for 1.5 h. Figure 11 shows no detectable pyrrhotite diffraction peaks, also confirming complete dissolution under these conditions.

**Figure 12:**
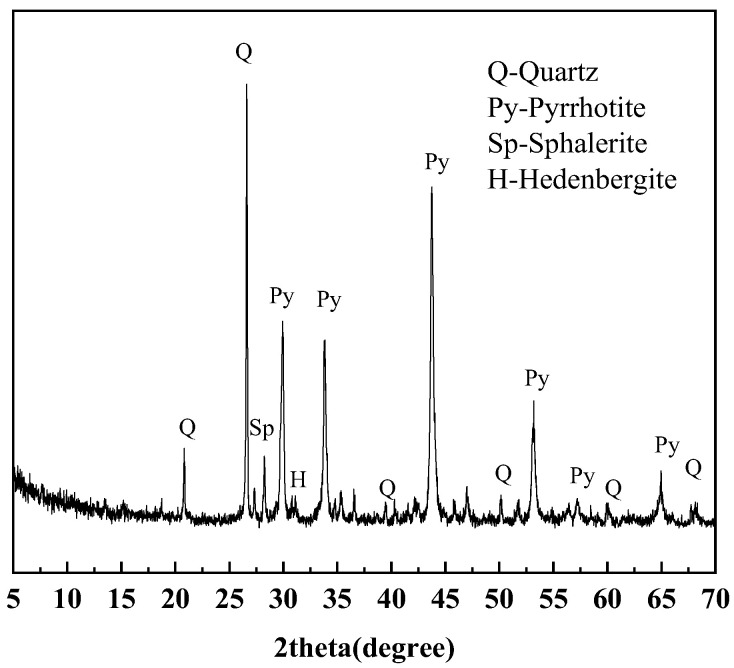
XRD pattern of pyrrhotite reacting with 1:1 HCl solution at 20 °C for 1.5 h.

When the temperature was reduced to 20 °C, Figure 12 shows that pyrrhotite did not fully react.

For an accurate quantitative analysis of pyrrhotite in practical samples, complete dissolution can be achieved by grinding the sample to all pass a 0.075 mm sieve, followed by a reaction with a 1:1 HCl solution at 50 °C for 1.5 h.

### 3.2. Quantification of Pyrite

**Figure 13:**
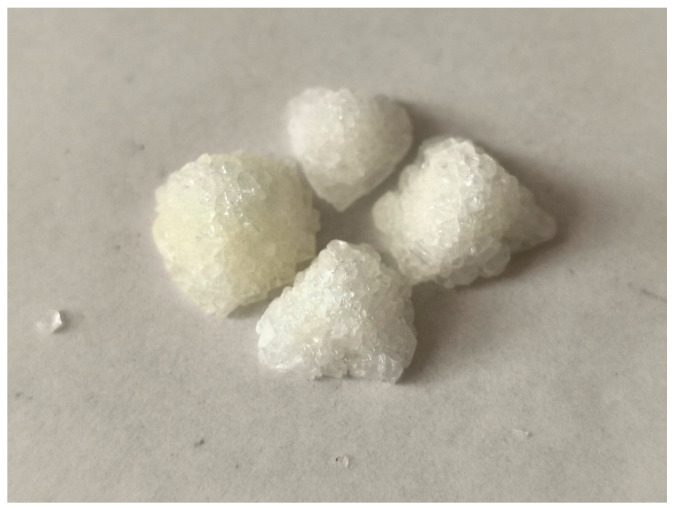
The boiling step caused the formation of Ba(NO_3_)_2_ precipitate.

Under the nine experimental conditions of the orthogonal design table, approximately 0.5 g of the sample was weighed (accuracy: ±0.0001 g) and reacted with 50 mL of acids (HNO_3_) at varying concentrations. Dilute the filtrate to 100 mL, take 5 mL of the filtrate, and add the BaCl_2_ solution to test for SO_4_^2−^. Table 8 shows the experimental results are presented. During the quantification of SO_4_^2−^ ions, the BaCl_2_ solution is added to the boiling solution, resulting in the formation of Ba(NO_3_)_2_ precipitate after standing. Originally, boiling was intended to promote the crystallization of barium sulfate precipitate, but it resulted in the formation of barium nitrate precipitate. Figure 13 shows boiling can lead to the formation of Ba(NO_3_)_2_ precipitate. Ba(NO_3_)_2_ has a stable crystalline form and is very difficult to dissolve in water and acid, with the result being on the high side. At room temperature, adding a BaCl_2_ solution, letting it stand for 4 h, and washing the precipitate with warm water can avoid the effect of Ba(NO_3_)_2_ on the results.

The pyrite content was quantified based on the calculated mass of sulfur in individual components, as shown in Equations (6) and (7).

(6) $$m_{total\;S}=m_{elemental\;S}+m_{Sin\;{SO}_{4}^{2-}}$$

(7) $$w_{{FeS}_{2}}=\frac{m_{total\;S}\times 1.87}{m_{sample}}$$

where $M_{FeS_{2}}=119.97\;g/mol$ and $M_{S}=32.06\;g/mol$. This yields a conversion factor of 1.87 for transforming the sulfur mass to a pyrrhotite mass.

The results in Table 8 indicate that, at 20 °C, with HNO_3_ concentrations of 1:1 and 2:1, the reaction between pyrite and HNO_3_ primarily produces SO_4_^2−^ ions, while a small amount of elemental sulfur is also formed. The elemental sulfur formed is mainly retained in the precipitate. When the HNO_3_ concentration is 3:1, pyrite (0.015–0.030 mm) reacts completely to form SO_4_^2−^ ions, with no elemental sulfur generated. As the reaction temperature increases to 50 °C and 80 °C, the elemental sulfur is further oxidized, resulting in no elemental sulfur, and all sulfur in the pyrite is converted to SO_4_^2−^ ions in the liquid phase. In the seventh experiment, the complete conversion of sulfur to SO_4_^2−^ ions was achieved even at 20 °C, primarily due to the high HNO_3_ concentration (3:1) and the small particle size (0.015–0.030 mm) of pyrite, which, together, facilitated the oxidation of elemental sulfur at relatively low temperatures. Therefore, increasing the HNO_3_ concentration and reducing the particle size of pyrite effectively promote the conversion of elemental sulfur at low temperatures, ensuring that sulfur exists entirely as SO_4_^2−^ ions in the liquid phase and reducing the need for additional quantitative steps.

Pyrite undergoes a complete reaction under standard conditions (particle size of 0.015–0.075 mm, temperature of 20–80 °C, HNO_3_ concentration exceeding 50%, and reaction time over 0.5 h). Significantly lower results are observed only under extreme conditions (particle size of 0.045–0.075 mm, 20 °C, dilute HNO_3_ 1:1, and a reaction time of 0.5 h, as demonstrated in Experiment 1). To ensure a complete reaction, avoid combinations of extreme parameters (e.g., coarse particles, low temperature, dilute acid, and a short reaction time). Adjusting any single parameter (e.g., reducing the particle size, increasing the temperature, using more concentrated HNO_3_, or extending the reaction time) is sufficient to achieve full reaction.

**Figure 14:**
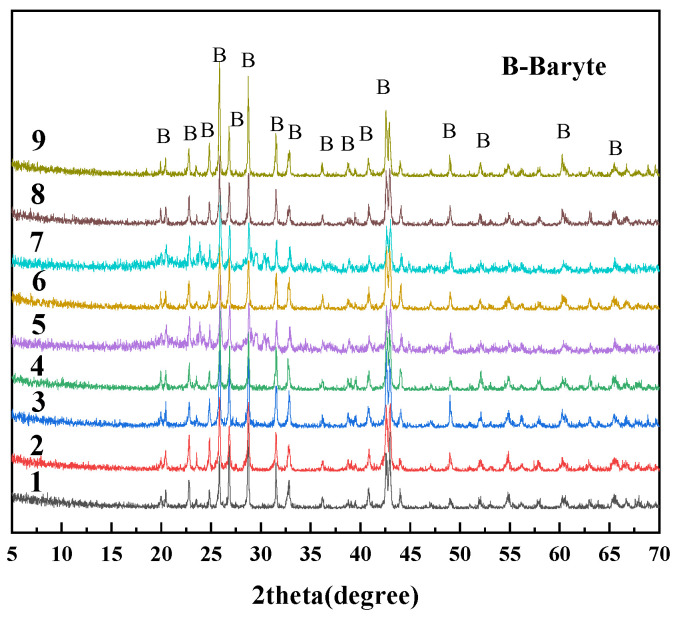
BaSO_4_ precipitate after incinerating at 950 °C.

Figure 14 shows that the precipitate is BaSO_4_, with no impurities such as Ba(NO_3_)_2_ detected.

Within the defined parameter range in Table 9, R4 > R1 > R2 > R3. The significance of the four factors is ranked as follows: the reaction time, sample particle size, reaction temperature, and HNO_3_ concentration. The reaction time and sample particle size have the greatest impact on the quantitative results of pyrite, followed by the reaction temperature, while the effect of the HNO_3_ concentration is minimal. The optimal conditions are A3B3C2D2, corresponding to a particle size of 0.015–0.030 mm, reaction temperature of 80 °C, HNO_3_ concentration of 2:1, and reaction time of 1 h.

The results indicate that, at a temperature range of 20–80 °C, particle sizes of 0.015–0.075 mm, a reaction time of over 0.5 h, and an HNO_3_ concentration exceeding 50%, the quantitative analysis of pyrite is accurate. However, when measuring tailings samples, the particle size distribution may not be as distinct. Therefore, it is necessary to test whether the optimal experimental conditions remain reasonable under a particle size range of below 0.075 mm and make further optimizations accordingly.

**Figure 15:**
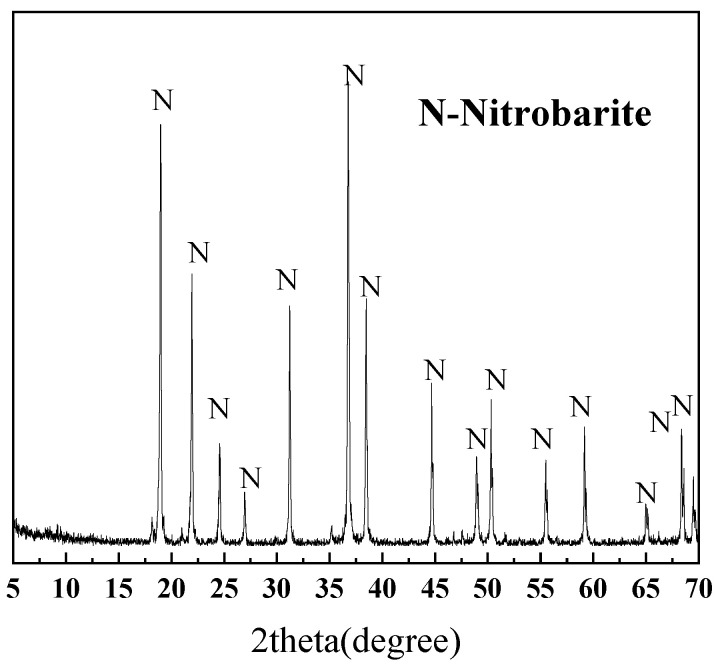
XRD pattern of the white precipitate after redissolution.

The pyrite completely dissolves after reacting with the 2:1 HNO_3_ solution at 80 °C for 1 h. Further optimization of the reaction conditions is recommended, under the two tested conditions: (1) a reaction temperature of 50 °C, a 2:1 HNO_3_ solution, and a reaction time of 1 h, and (2) a reaction temperature of 80 °C, a 1:1 HNO_3_ solution, and a reaction time of 1 h. Due to the small amount of filter residue, the X-ray diffraction analysis pattern is not feasible. The filter residue was ground and redissolved in a concentrated 2:1 HNO_3_ solution at 80 °C to verify the complete pyrite dissolution by quantifying SO_4_^2−^ ions in the resulting filtrate. Figure 15 shows it is Ba(NO_3_)_2_. No characteristic peaks of barium sulfate are detected, confirming that the pyrite in the sample is completely decomposed under the tested conditions.

**Figure 16:**
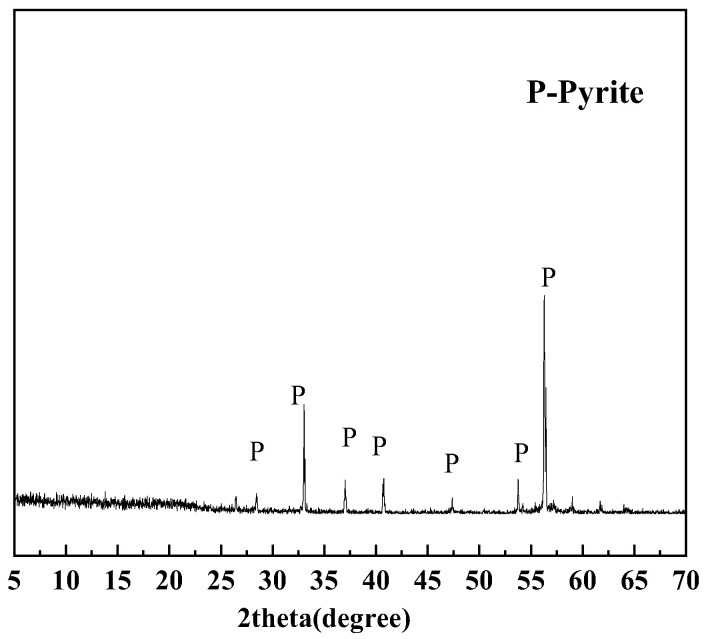
XRD pattern of pyrite reacting with 1:1 HNO_3_ solution at 50 °C for 1 h.

Further optimization of the reaction conditions: Pyrite is reacted with a 1:1 HNO_3_ solution at 50 °C for 1 h. Figure 16 shows significant pyrite characteristic diffraction peaks in the residue, indicating that the pyrite did not fully react.

For an accurate quantitative analysis of pyrite in practical samples, complete dissolution can be achieved by grinding the sample to below 0.075 mm, followed by a reaction with a 1:1 HNO_3_ solution at 80 °C for 1 h.

### 3.3. Quantification of Iron Sulfide in Iron Ore Tailings

#### 3.3.1. Quantification with Chemical Dissolution Methods

Iron ore tailings samples were first dissolved in HCl. Quantitative pyrrhotite was reacted with 1:1 HCl at 50 °C for 1.5 h. The residue was then dissolved in carbon disulfide, and the filtrate was used to distill elemental sulfur. After drying the residue, HNO_3_ was added, and the mixture was reacted with a 1:1 HNO_3_ solution at 80 °C for 1 h. The resulting filtrate was diluted to 100 mL. From this, 5 mL was taken and mixed with the excess BaCl_2_ solution, left to stand for at least 4 h, washed with hot water 8 to 10 times, and then ashed at 350 °C, followed by calcination at 950 °C. Table 10 shows the quantitative results of sulfide iron and soluble sulfate in the samples.

#### 3.3.2. Quantification with the Internal Standard Method of XRD

The quantitative analysis using the internal standard method determined the pyrite content as follows: 2.77% in crushed stone sample 1, 1.10% in crushed stone sample 2, 6.13% in crushed stone sample 3, 5.95% in manufactured sand sample 4, 1.87% in manufactured sand sample 5, and 2.63% in manufactured sand sample 6.

**Figure 17:**
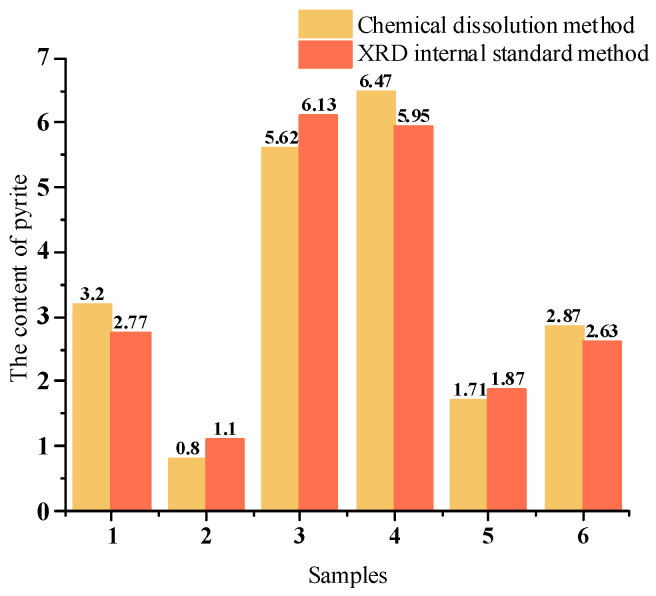
Quantitative results of pyrite.

Figure 17 shows that chemical dissolution approach achieves comparable pyrite content measurement (deviation <0.6%) to the internal standard method of XRD. The selected samples are representative. The deviation in results for Sample 1 is +0.43%, Sample 2 is −0.3%, Sample 3 is −0.51%, Sample 4 is +0.52%, Sample 5 is −0.16%, and Sample 6 is +0.24%.

## 4. Discussion

### 4.1. Optimization of Experimental Conditions

The initial experimental design using three particle size ranges (0.015–0.030 mm, 0.030–0.045 mm, and 0.045–0.075 mm) provided a detailed characterization of individual minerals. Industrial applications require more cost-effective and practical conditions. To address this, we optimized the protocol by (1) setting the particle size to <0.075 mm, and (2) reducing the reaction temperature for pyrrhotite from 80 °C to 50 °C and decreasing the HNO_3_ concentration for pyrite from 2:1 to 1:1, significantly lowering the energy and reagent consumption. The test results confirmed complete reactions under the optimized conditions, enabling a wider field application of this technique.

### 4.2. Accuracy and Precision of Chemical Dissolution Methods

For pyrrhotite quantification, orthogonal tests 1, 2, 3, 4, and 7 showed that the quantitative analysis could not be effectively completed under the current experimental conditions. Under suitable conditions (tests 5, 6, 8, and 9), the average quantitative result was 84.26% with a standard deviation of 3.03 and a relative standard deviation (RSD) of 3.60% (<5%), showing a good repeatability and high precision. The absolute errors were 3.47, 2.00, 1.40, and 4.13, with relative errors of 4.07%, 2.34%, 1.60%, and 4.73%, respectively. The mean absolute error (MAE) was 2.75 and mean relative error (MRE) was 3.19% (<5%), showing a good accuracy and agreement with reference values.

For pyrite quantification under proper conditions (excluding test 1), the average measured value was 93.53%, with a standard deviation of 2.91 and a relative standard deviation of 3.11% (<5%), showing good repeatability and high precision. The mean absolute error was 4.17 and mean relative error was 4.70% (<5%), showing a high accuracy and good agreement with reference values.

For mineral samples, the pyrite content determined by chemical methods ranged from 0.8% to 6.47%, while XRD with the internal standard method yielded 1.1% to 6.13%. The error between these methods varied between −0.51% and +0.52%.

The method demonstrates high precision, repeatability, and accuracy in the quantitative analysis of both iron sulfide minerals (pyrrhotite and pyrite) under suitable conditions, showing a good agreement with the XRD internal standard method, making it a reliable analytical technique.

### 4.3. Method Comparisons

The chemical dissolution method proposed in this study complements various analytical techniques reported in the literature (e.g., EDS, XRD, synchrotron XRF, and S-XANES) in terms of objectives, applicability, and limitations. Luo et al. [25] achieved a quantitative analysis of pyrite thin specimens using EDS, but this method requires stringent sample preparation (thin specimens) and struggles to distinguish the dissolution selectivity between pyrrhotite and pyrite. Mcdougall et al. [26] employed XRD for the quantitative phase analysis, which can differentiate between mineral phases but relies on standard samples and lacks sensitivity for low-content phases. Del Real et al. [27] combined multiple microanalytical techniques (e.g., synchrotron XRF) for elemental mapping, providing a high spatial resolution, but the equipment is costly and unsuitable for batch sample processing. Bolin [28] used S-XANES to directly determine the pyrite content, which is sensitive to sulfur speciation but requires synchrotron radiation, limiting its application in conventional laboratories.

In contrast, the chemical dissolution method in this study enables the differentiation and quantification of multi-phase sulfides by optimizing the acid system, allowing the simultaneous quantitative analysis of soluble sulfates, pyrrhotite, and pyrite. However, a limitation of the chemical method is its susceptibility to interference from associated minerals (e.g., other sulfides or oxides).

## 5. Conclusions

In this study, an on-site quantification procedure was proposed for determining the pyrite and pyrrhotite content in tailings using a selective chemical dissolution process. Sulfur-containing products were quantitatively analyzed, and the iron sulfide content was calculated based on chemical formulae, excluding the interference of galena and lead sulfide in the quantification process.

(1) The optimal quantitative conditions were determined as grinding pyrrhotite to 0.045–0.075 mm, followed by digestion in 1:1 HCl at 80 °C for 1.5 h, while pyrite required finer grinding to 0.015–0.030 mm with a subsequent treatment in 2:1 HNO_3_ at 80 °C for 1 h to achieve complete dissolution.
(2) The pyrrhotite quantification method exhibited a relative standard deviation (RSD) of 3.60% (<5%) and a mean relative error of 3.19% (<5%), while the pyrite quantification method showed an RSD of 3.11% (<5%) with a mean relative error of 4.70% (<5%).
(3) The dissolution process was improved, the cost was reduced, and on-site well completion was achieved. The quantitative results of pyrite in mineral samples were verified by the XRD internal standard method, and the error was less than 0.6%.

## Figures and Tables

**Table 1:** Elemental compositions of pyrrhotite in 0.015–0.030 mm, 0.030–0.045 mm, and 0.045–0.075 mm.

Particle Size/mm	Elemental Composition/wt%							
	Fe	S	Pb	Zn	Si	Cu	Sb	Al
0.015–0.030	58.42	33.84	2.23	0.95	0.49	0.20	0.19	0.11
0.030–0.045	60.33	34.18	0.95	0.47	0.39	0.09	0.07	0.05
0.045–0.075	59.19	35.15	1.36	0.72	0.37	0.15	0.12	0.07

**Table 2:** Elemental compositions of pyrite in 0.015–0.030 mm, 0.030–0.045 mm, and 0.045–0.075 mm.

Particle Size/mm	Elemental Composition/wt%							
	S	Fe	Si	Ca	W	Mg	Al	Cl
0.015–0.030	48.79	47.05	0.57	0.30	0.18	0.10	0.04	0.03
0.030–0.045	46.62	49.62	0.48	0.23	0.11	0.06	0.03	0.03
0.045–0.075	47.95	49.06	0.44	0.19	0.10	0.05	0.03	0.03

**Table 3:** Determination of pyrite content in samples by XRD internal standard method.

Sample	Percentage/%				
Albite	94	93	91	89	87
Pyrite	1	2	4	6	8
ZnO	5	5	5	5	5

**Table 4:** Orthogonal experimental design table for reaction between pyrrhotite and HCl.

Level	A Particle Size/mm	B Temperature/°C	C HCl Concentration	D Time/h
1	0.015–0.030	20	1:1	1.5
2	0.030–0.045	50	1:3	2.0
3	0.045–0.075	80	1:5	2.5

**Table 5:** Orthogonal experimental design table for reaction between pyrite and HNO3.

Level	A Particle Size/mm	B Temperature/°C	C HNO_3_ Concentration	D Time/h
1	0.045–0.075	20	1:1	0.5
2	0.030–0.045	50	2:1	1.0
3	0.015–0.030	80	3:1	1.5

**Table 6:** Experimental results of pyrrhotite.

Experiment	A	B	C	D	Sample/g	Elemental Sulfur/g	CuS/g	Fe_7_S_8_/%
1	A1 *	B1	C1	D1	0.4997	0.0120	0.1653	33.15
2	A1	B2	C2	D2	0.5007	0.0189	0.1067	26.41
3	A1	B3	C3	D3	0.4873	0.0250	0.3520	72.91
4	A2	B1	C2	D3	0.5051	0.0000	0.0000	0.00
5	A2	B2	C3	D1	0.5340	0.0239	0.4276	81.85
6	A2	B3	C1	D2	0.4974	0.0184	0.4867	83.32
7	A3	B1	C3	D2	0.4995	0.0000	0.0000	0.00
8	A3	B2	C1	D3	0.5041	0.0261	0.4611	88.70
9	A3	B3	C2	D1	0.5000	0.0295	0.4026	83.17

* A1 represents factor A at level 1.

**Table 7:** Orthogonal experimental intuitive analysis of pyrrhotite.

Experiment	A	B	C	D	The Reacted Fe_7_S_8_/%	Reference Value/%	Ratio/%
1	1	1	1	1	33.15	83.32	60.21
2	1	2	2	2	26.41	83.32	68.30
3	1	3	3	3	72.91	83.32	12.50
4	2	1	2	3	0.00	85.32	100.00
5	2	2	3	1	81.85	85.32	4.07
6	2	3	1	2	83.32	85.32	2.34
7	3	1	3	2	0.00	87.30	100.00
8	3	2	1	3	88.70	87.30	1.60
9	3	3	2	1	83.17	87.30	4.73
k1	47.00	86.74	21.38	23.00			
k2	35.47	24.66	57.68	56.88			
k3	35.44	6.52	38.86	38.03			
Range	11.56	80.21	36.29	33.88			
Optimization Plan	A3	B3	C1	D1			

**Table 8:** The content of pyrite measured by barium sulfate gravimetric method.

Experiment	A	B	C	D	Sample/g	Elemental Sulfur/g	BaSO_4_/(g/5 mL)	FeS_2_/%
1	A1	B1	C1	D1	0.5120	0.0032	0.0721	73.57
2	A1	B2	C2	D2	0.5084	0.0000	0.0933	94.35
3	A1	B3	C3	D3	0.5067	0.0000	0.0949	96.29
4	A2	B1	C2	D3	0.4966	0.0025	0.0906	94.74
5	A2	B2	C3	D1	0.4982	0.0000	0.0939	96.90
6	A2	B3	C1	D2	0.5020	0.0000	0.0857	87.77
7	A3	B1	C3	D2	0.5082	0.0000	0.0906	91.66
8	A3	B2	C1	D3	0.5064	0.0000	0.0911	92.49
9	A3	B3	C2	D1	0.5008	0.0000	0.0916	94.04

**Table 9:** Orthogonal experimental results and analysis of pyrite.

Experiment	A	B	C	D	The Reacted FeS_2_/%	Reference Value/%	Ratio/%
1	1	1	1	1	73.57	89.71	17.99
2	1	2	2	2	94.35	89.71	5.18
3	1	3	3	3	96.29	89.71	7.34
4	2	1	2	3	94.74	87.22	8.62
5	2	2	3	1	96.90	87.22	11.10
6	2	3	1	2	87.77	87.22	0.63
7	3	1	3	2	91.66	91.28	0.41
8	3	2	1	3	92.49	91.28	1.33
9	3	3	2	1	94.04	91.28	3.02
k1	10.17	9.01	6.65	10.70			
k2	6.78	5.87	5.61	2.07			
k3	1.59	3.66	6.28	5.76			
Range	8.58	5.34	1.04	8.63			
Optimization Plan	A3	B3	C2	D2			

**Table 10:** Quantitative results of soluble sulfate and iron sulfide.

Sample	Soluble Sulfates (in Terms of SO_3_/%)	Fe_1−x_S (in Terms of Fe_1−x_S Mass/%)	FeS_2_ (in Terms of FeS_2_ Mass/%)
1	0.09	0.00	3.20
2	0.10	0.00	0.80
3	0.09	0.00	5.62
4	0.10	0.00	6.47
5	0.12	0.00	1.71
6	0.10	0.00	2.87

## Data Availability

The original contributions presented in this study are included in the article. Further inquiries can be directed to the corresponding authors.

## References

[1] Saedi A., Jamshidi-Zanjani A. (2025). Addition of silane nanoparticles combined with mechanical activation for efficient reuse of sulfidic tailings for concrete construction. Dev. Built Environ..

[2] Li L.J., Mao X.C., Liu Z.K., Wang Y.C., Li D.X., Ai Q.X., Wang Y.Q. (2024). Variation of chalcophile elements in base metal sulfide minerals from the Jinchuan magmatic Ni—Cu sulfide deposit, NW China: Implications for mineral exploration. J. Geochem. Explor..

[3] Li S., Xiao W., Chen C., Sang M., Mao Q., Gao L., Xia F., Li X., Du X. (2024). Two-episode mineralization in the Haerdaban Pb—Zn deposit, NW China: Insights from sulfide trace elements, in situ S—Pb isotopes, and Rb-Sr geochronology. Precambrian Res..

[4] Wang Y., Lai J., Cao Y., Brzozowski M., Mao X., Peng H., Ai Q. (2023). Controls on the metal tenors of sulfide ores in the Jinchuan Ni-Cu-PGE sulfide deposit, NW China: Implications for the formation of distinct textural types of sulfide ores in magma conduits. Ore Geol. Rev..

[5] Zhong R., Wille K. (2018). Deterioration of residential concrete foundations: The role of pyrrhotite-bearing aggregate. Cem. Concr. Compos..

[6] Glasser F.P., Marchand J., Samson E. (2008). Durability of concrete—Degradation phenomena involving detrimental chemical reactions. Cem. Concr. Res..

[7] Rodrigues A., Duchesne J., Fournier B., Durand B., Rivard P., Shehata M. (2012). Mineralogical and chemical assessment of concrete damaged by the oxidation of sulfide—Bearing aggregates: Importance of thaumasite formation on reaction mechanisms. Cem. Concr. Res..

[8] Schmidt T., Leemann A., Gallucci E., Scrivener K. (2011). Physical and microstructural aspects of iron sulfide degradation in concrete. Cem. Concr. Res..

[9] Jana D. (2022). Cracking of residential concrete foundations in eastern Connecticut, USA from oxidation of pyrrhotite. Case Stud. Constr. Mater..

[10] Marcelino A.P., Calixto J.M., Gumieri A.G., Caldeira C.L., Delbem I.D., Ferreira M.C. (2020). A feasible evaluation protocol to determine the most reactive sulfide-bearing aggregate for use in concrete. Constr. Build. Mater..

[11] Strongman K.R., Gibson H.L., Howard A.E., Lafrance B., Hamilton M.A. (2025). Relative timing and controls on advanced argillic and conventional alteration of the Neoarchean Onaman volcanogenic massive sulfide deposit, Ontario, Canada. Can. J. Earth Sci..

[12] Titon B., Duchesne J., Fournier B., Rodrigues A. (2025). Concrete deterioration due to sulfide-bearing aggregates: Mineralogical and chemical characteristics of pyrrhotite in concrete aggregates from Trois-Rivières, Canada. Constr. Build. Mater..

[13] Jana D. (2020). Pyrrhotite Epidemic in Eastern Connecticut: Diagnosis and Prevention. Aci Mater. J..

[14] Fan Z., Zou D., Zhang M., Qin S., Liu T. (2025). Numerical modeling of unidirectional sulfate attack on tunnel lining concrete considering water evaporation at free face. Cem. Concr. Res..

[15] Nehdi M.L., Suleiman A.R., Soliman A.M. (2014). Investigation of concrete exposed to dual sulfate attack. Cem. Concr. Res..

[16] Ragoug R., Metalssi O.O., Barberon F., Torrenti J.M., Roussel N., Divet L., de Lacaillerie J.B.D.E. (2019). Durability of cement pastes exposed to external sulfate attack and leaching: Physical and chemical aspects. Cem. Concr. Res..

[17] Andrews A., Nyarko E.F., Adjaottor A.A., Nsiah-Baafi E., Adom-Asamoah M. (2022). Reuse and stabilization of sulphide mine tailings as fine aggregate for construction mortar. J. Clean. Prod..

[18] Rimstidt J., Vaughan D.J. (2003). Pyrite oxidation: A state-of-the-art assessment of the reaction mechanism. Geochim. Cosmochim. Acta.

[19] Janzen M.P., Nicholson R.V., Scharer J.M. (2000). Pyrrhotite reaction kinetics: Reaction rates for oxidation by oxygen, ferric iron, and for nonoxidative dissolution. Geochim. Cosmochim. Acta.

[20] Hamilton I.C., Woods R. (1981). An investigation of surface oxidation of pyrite and pyrrhotite by linear potential sweep voltammetry. J. Electroanal. Chem..

[21] Oliveira I., Cavalaro S.H., Aguado A. (2013). New kinetic model to quantify the internal sulfate attack in concrete. Cem. Concr. Res..

[22] Wang H., Dowd P.A., Xu C. (2019). A reaction rate model for pyrite oxidation considering the influence of water content and temperature. Miner. Eng..

[23] Chirita P., Schlegel M.L. (2017). Pyrite oxidation in air-equilibrated solutions: An electrochemical study. Chem. Geol..

[24] Belzile N., Chen Y.W., Cai M.F., Li Y. (2004). A review on pyrrhotite oxidation. J. Geochem. Explor..

[25] Luo T., Guo Y., Deng Z., Liu X., Sun Z., Qi Y., Yang M. (2023). Energy-dispersive X-ray spectroscopy for the quantitative analysis of pyrite thin specimens. J. Wuhan Univ. Technol.-Mater. Sci. Ed..

[26] Mcdougall H., Hibberd M., Tong A., Neville S., Peterson V., Didier C. (2022). Preparation of pyrite concentrate powder from the Thackaringa mine for quantitative phase analysis using X-ray diffraction. J. Appl. Crystallogr..

[27] Del Real I., Smieska L., Thompson J.F.H., Martinez C., Thomas J., Layton-Matthews D. (2019). Using multiple micro-analytical techniques for evaluating quantitative synchrotron-XRF elemental mapping of hydrothermal pyrite. J. Anal. At. Spectrom..

[28] Bolin T.B. (2010). Direct Determination of Pyrite Content in Argonne Premium Coals by the Use of Sulfur X-ray Near Edge Absorption Spectroscopy (S-XANES). Energy Fuels.

[29] (2014). Iron Tailings Sand.

[30] (2008). Aggregates for Concrete.

[31] (2016). Guide to Durable Concrete.

